# Individual response in basal metabolism and nutritional composition to asexual reproduction in tropical sea cucumber *Holothuria atra*

**DOI:** 10.7717/peerj.21358

**Published:** 2026-06-02

**Authors:** Haiqing Wang, Yan Zhao, Jingting Xie, Yanchao Chai

**Affiliations:** Hainan University, Haikou, China

**Keywords:** *Holothuria atra*, Asexual reproduction, Metabolism, Energy, Fatty acids

## Abstract

**Backgroud:**

Asexual reproduction *via* fission plays a vital role in the survival of the sea cucumber *Holothuria atra*. However, the energetic and nutritional mechanisms driving its regenerative capacity remain poorly understood.

**Method:**

Fission was induced in the sea cucumber *Holothuria atra* by constricting the mid-body with a rubber band. Following fission, the resulting fragments were designated as anterior (A) and posterior (P) based on their original body orientation. The growth, oxygen consumption, ammonium (NH_4_^+^) excretion rates and biochemical composition were then assessed in both anterior and posterior fragments at 10, 20, 30, 40, 50 and 60 days post-fission regeneration.

**Results:**

Induced fission generated anterior and posterior fragments. Both fragments exhibited significant weight loss, with a 31.6% reduction observed by day 10. Routine metabolic rates (RMR) were elevated during regeneration, particularly in anterior fragments, which peaked at 1.06 ± 0.46 mg O_2_ h^−1^ g^−1^ _DW (dry body weight)_ by day 40. Simultaneously, increased ammonia excretion rates indicated a reliance on protein catabolism as a primary energy source (O:N ratio < 10). Despite these metabolic shifts, the proximate biochemical composition (levels of protein, lipid and carbohydrate) remained largely stable during the regeneration. An exception was a transient peak in specific fatty acids (*e.g.*, C18:1n-9, MUFA) at day 30, which may be associated with membrane synthesis. Moreover, posterior fragments showed higher carbohydrate mobilization by day 50 (*p* < 0.05).

**Conclusion:**

These findings suggest that *H. atra* may maintain homeostasis during fission by modulating metabolism rather than depleting stored nutrients, possibly entering a hypometabolic state to conserve energy. This study highlights the resilience of *H. atra* to asexual reproduction stressors and underscores the need for further research on exogenous nutrient absorption during regeneration.

## Introduction

Sea cucumbers are vital benthic organisms in tropical coral reef ecosystems, and they play an indispensable role in sediment purification, nutrient cycling, and ecosystem stability (Uthicke & Conand, 2024). By digesting organic matter from carbonate sediments, they enhance reef alkalinity, mitigate ocean acidification, and promote coral growth (Clements et al., 2024; Schneider et al., 2013; Wolfe et al., 2018). *Holothuria atra* is commonly distributed in the tropical Indo-Pacific region. They used to live in sandy sediment with coral sand, sifting through the sediment with its tentacles and feeding on detritus and other organic matter (Viyakarn et al., 2020; Wolfe & Davey, 2020). An individual *H. atra* is estimated to produce approximately 14 kg of bioturbated sediment per year (Williamson et al., 2021). Therefore, stable sea cucumber populations are critical in keeping coral reef healthy. Additionally, *H. atra* possesses significant biomedical value, with demonstrated antioxidant and anticancer properties (Chao, Chen & Alexander, 1993; Nugroho et al., 2022), further emphasizing the importance of understanding its reproductive physiology.

Asexual reproduction is a key strategy for population replenishment in tropical sea cucumbers, particularly in species such as *H. atra*, *Stichopus chloronotus* and *H. edulis* (Benjamin, Hampus & Maria, 2012; Dolmatov, Khang & Kamenev, 2012; Uthicke & Conand, 2024). *H. atra* exhibits year-round fission with seasonal variations in frequency (Chao, Chen & Alexander, 1993). The parameters like high mortality, low habitat stability, a small optimal body size, abundant food, and limited larval supply drive asexual reproduction *via* fission in these locations (Lee, Byrne & Uthicke, 2008; Sonnenholzner, Searcy-Bernal & Panchana-Orrala, 2017; Uthicke & Conand, 2024). The regeneration process following fission presents significant challenges, including asymmetric organ retention between anterior and posterior segments.

However, post-fission survival remains controversial, with conflicting reports on whether anterior or posterior segments regenerate more successfully (Conand, 1997; Pattinasarany & Pattiasina, 2020; Thorne & Byrne, 2013). This discrepancy may stem from differences in residual organ function and nutrient allocation, highlighting the need to investigate metabolic utilization patterns during regeneration. High variability in regeneration growth, and survival rates, all of which may be closely linked to nutrient availability and energy metabolism (Dolmatov, 2014a; Dolmatov, 2014b; Dolmatov, Khang & Kamenev, 2012; Lawrence, 2010; Thorne & Byrne, 2013). However, the mechanisms governing nutrient allocation during asexual reproduction remain poorly understood. Therefore, elucidating the nutrient absorption strategies in regenerating sea cucumbers is critical for advancing asexual reproduction technologies and ensuring their ecological sustainability.

The energetic demands of asexual reproduction are substantial, involving not only tissue regeneration but also metabolic costs during non-feeding periods (Lawrence, 2010). While 18 species of sea cucumbers exhibit regenerative capabilities, only a few tropical species, such as *H. atra*, *H. edulis*, *H. leucospilota* and *H. hilla*, rely on fission as a primary reproductive strategy (Dolmatov, Khang & Kamenev, 2012; Lawrence, 2010; Wolfe & Davey, 2020). Regeneration requires both structural rebuilding and sustained energy supply, but how sea cucumbers balance endogenous reserves and exogenous nutrient uptake remains unclear. Previous studies on echinoderms suggest that stored nutrients or dissolved organic matter absorption may support regeneration (Brothers, Lee & Nestler, 2015; Dobson et al., 1991), but no such mechanism has been confirmed in asexually reproducing sea cucumbers.

Current research on sea cucumber regeneration primarily focuses on visceral regeneration after evisceration, revealing reliance on endogenous energy reserves (Sun et al., 2017). Observations in *H. atra* show initial weight loss post-fission, followed by gradual recovery (Pattinasarany & Pattiasina, 2020), suggesting dynamic nutrient mobilization. However, whether exogenous nutrient absorption compensates for energy deficits during regeneration remains unexplored. Many marine invertebrates supplement regeneration through epidermal or respiratory uptake of dissolved organics, but similar mechanisms in sea cucumbers are unverified.

This study aims to characterize the biochemical composition shifts in *H. atra* during asexual reproduction, quantify nutrient budgets throughout regeneration, and identify the primary energy sources sustaining metabolism. By addressing these gaps, we seek to enhance asexual reproduction techniques and inform conservation strategies for coral reef ecosystems.

## Materials & Methods

### Experimental animals and condition

The sea cucumber *H. atra* were collected by SCUBA from Qionghai, Hainan Province. Around 70 black sea cucumbers with similar size (64.53 ± 14.99 g) were quickly transported to the laboratory and acclimated for seven days in a glass recirculation system (120 × 80 × 70 cm, 672 L). During the whole period, water temperature was maintained at 25 ± 1 °C, with a salinity of 29 ± 1 and continuous aeration. The sea cucumbers were fed daily with an artificial feed composed of *Sargassum thunbergii* and other *Sargassum* powder, formulated feed, and sea mud, in a 1:1:1:1 ratio. The daily feed amount was set at 6% of the sea cucumbers’ body weight. Feces and any uneaten feed were removed before feeding.

### Fission induced

**Figure 1:**
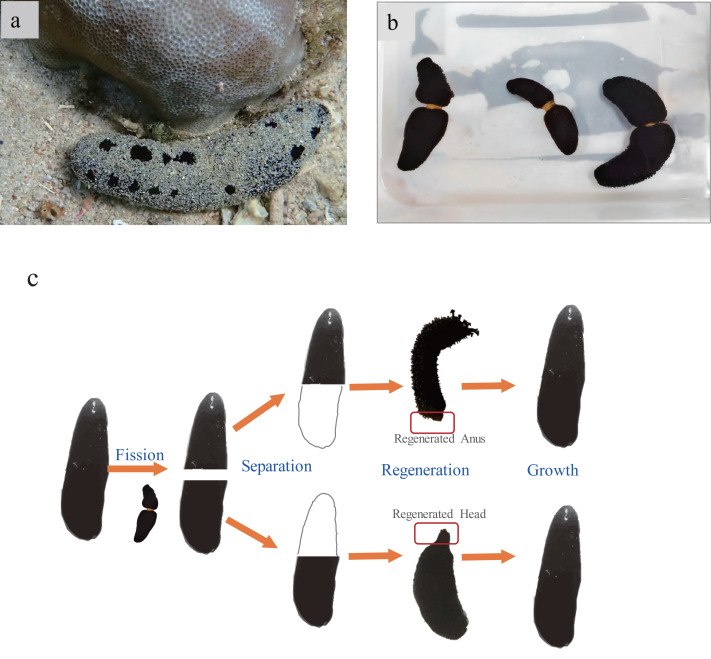
(A) Photograph of sea cucumber *Holothuria atra* living in a natural sand bed within a coral area (photoed by Chunyang Sun). (B) *H. atra* were induced transverse fission with rubber band. (C) Schematic diagram of fission and regeneration process. *H. atra* were induced transverse fission with rubber band.

Sea cucumbers *H. atra* underwent fission using rubber band. Rubber bands were applied to tighten the body of *H. atra* at the midpoint, 50% from the anterior, to promote rapid fission and regeneration (Fig. 1). The sea cucumber will then stretch and twist where the rubber band is fastened. Finally, the new individuals resulted from the fission plane were anterior (A) and posterior (P) when the body of sea cucumber was divided into two parts. The fission will most happen in 12 h, and the wound will gradually shrink. In order not to cause the wound deterioration, the first sampling was conducted 10 days after fission.

### Experimental conditions post fission

To ensure no cross-contamination or mix-up of the fission results, each individual sample was placed in a container. The anterior and posterior regions can be distinguished by the presence of tentacles on the anterior part. These containers were clearly labeled with unique identifiers (processing time) prior to the experiment. Since the new cloaca, head, and digestive tract required regeneration, no feeding was provided during the post-fission experimental period. Half of the seawater was replaced daily.

### Determination of oxygen consumption and NH_4_^+^ excretion rates

The oxygen consumption was carried out at 0, 10, 20, 30, 40, 50 and 60 days post-fission during regeneration. The sea cucumbers were allowed to acclimate until they adhered to the wall as the beginning. The respiration chamber was a 1.5 L brown glass bottle sealed with a rubber stopper. It was submerged in a temperature-controlled water bath (maintained at 25 °C) for the 1.5-hour incubation period. Dissolved oxygen concentrations were measured using fiber optic oxygen sensors (PreSens Oxy, Germany). At each time point, three anterior and three posterior sections were placed into separate respirometers. After the respiration measurement, each sea cucumber was removed and weighed. A portion of tissue from each specimen were used to determine water content, whereas the remaining portions were snap-frozen in liquid nitrogen and stored at −80 °C for subsequent nutritional composition analysis. Three respiratory chambers without the sea cucumbers were also monitored for detection of background respiration. The initial and final oxygen concentrations in the respiratory chamber were monitored, and confirm that levels remained above six mg/L by the end of the experiment to prevent the test organisms from being exposed to hypoxic conditions.

Ammonium excretion rates were determined from ammonium (NH_4_^+^) concentration measurements prior to and following the oxygen consumption. Before closing and after opening respiratory chambers, about five mL seawater were filtered through a 0.45 µm filter to measure ammonia concentration. NH_4_^+^ concentration was determined the sodium hypobromite oxidation method by spectrophotometry (Inspection and Quarantine of the People’s Republic of China and Standardization Administration, 2007).

The routine metabolic rate (RMR, mg O_2_ h^−1^ g^−1^_DW (dryweight)_), (1) and NH_4_^+^ excretion rate (AER, mg NH_4_^+^ h^−1^ g^−1^_DW_), (2) are calculated using the following equations:

(1) \begin{eqnarray*}\mathrm{RMR}& = \frac{({R}_{t}-{R}_{0})\times V}{W\times T} \end{eqnarray*}

(2) \begin{eqnarray*}\mathrm{AER}& = \frac{({R}_{t}-{R}_{0})\times V}{W\times T} \end{eqnarray*}

where, *R*_*t*_ is the oxygen and NH_4_^+^ concentration at each time point (mg/L), *R*_0_ is the oxygen and NH_4_^+^ concentration at the initial of the experiment (mg/L). V is the corrected respirometer volume (L), W is the dry weight of sea cucumber in one chamber (g), which is calculated based on the dry matter content of each treatment group. T is the duration of the sea cucumbers in the respiratory chamber (h).

Weight change rate (g/day) was calculated as the coefficient of the linear regression between wet weight (g) and regeneration time (d).

### Biochemical composition analysis

After measuring the rates of oxygen consumption and NH_4_^+^ excretion, three anterior and three posterior sections were dried, weighed, and subsequently dissected at each time point during the regeneration period (n = 3 per group per time). The moisture, ash and biochemical composition includes total protein, carbohydrate, lipid, fatty acids, and amino acids, of *H. atra* were analyzed during their regeneration period. The ash content was evaluated by burning at 550 °C for 6 h in a muffle furnace after dried in oven. Carbohydrate was determined with phenol-sulfuric acid method by spectrophotometry.

#### Protein and lipid

The crude protein contents were determined by Kjelahl method. Nitrogen content was analyzed with a Kjeldahl nitrogen analyzer. The nitrogen content multiplied 6.25 was used to convert nitrogen content into protein content.

The total lipid content was determined gravimetrically by weighing an aliquot of extract in a pre-weighed vial. The crude fat content of the sample was digested with hydrochloric acid in a water bath and then extracted with ethanol and anhydrous ether. The supernatant was transferred into a pre-weighed vial and dried at 100 ±5 °C for 1 h. Finally, the vial was weighed after cooling.

#### Amino acids analysis

The amino acid compositions of the sea cucumber tissues were analyzed following the method described by Lin et al. (2018). Weigh an appropriate amount of the homogenized sample into a hydrolysis tube. Then, five mL of a 1:1 hydrochloric acid solution was added and mixed thoroughly. The tube was placed in an electric forced-air oven at 110 °C ± 1 °C for hydrolysis over 22 h. After hydrolysis, the tube was removed and cooled to room temperature. The hydrolysate was filtered into a 10 mL volumetric flask. The hydrolysis tube was rinsed several times with a small amount of water, and the rinsates were combined into the same flask. The solution was then diluted to the mark with water and mixed well. Subsequently, 0.5 mL of the filtrate was accurately transferred to a 15 mL test tube and dried under a stream of nitrogen. The residue was reconstituted in two mL of 0.02 mol/L hydrochloric acid solution, mixed thoroughly, filtered through a 0.22 µm membrane filter, and finally analyzed using an automatic amino acid analyzer (LA8080, Hitachi, Japan) with a sulfonic acid cationic resin column. The mobile phase ran at a flow rate of 0.35 mL/min, and the column temperature was maintained at 135 ± 5 °C. The reaction liquid flow rate was set at 0.3 mL/min, with wavelength adjustment from 570 nm to 440 nm.

#### Fatty acids analysis

An appropriate amount of well-mixed sample was weighed into a flask. About 100 mg of pyrogallic acid and several boiling stones were added, followed by two mL of 95% ethanol. The mixture was homogenized, and 10 mL of hydrochloric acid solution was then added and mixed thoroughly. The flask was placed in a water bath maintained at 70–80 ^∘^C and hydrolyzed for 40 min. During hydrolysis, the flask was swirled every 10 min to redissolve any particles adhering to the walls. After hydrolysis, the flask was removed and allowed to cool to room temperature.

To the hydrolyzed sample, 10 mL of 95% ethanol was added and mixed. The hydrolysate was transferred into a separatory funnel. The flask and stopper were rinsed with 50 mL of an ether-petroleum ether mixture, and the rinsate was combined into the same separatory funnel. After capping, the funnel was shaken vigorously for 5 min and then left to stand for 10 min. The ether layer was collected into a 250 mL flask. This extraction procedure was repeated three times. Finally, the separatory funnel was rinsed with the ether-petroleum ether mixture, and the rinsate was collected into a pre-weighed flask. The flask was placed on a water bath to evaporate the solvent, then dried in an oven at 100 ± 5 ^∘^C for 2 h.

Fatty acids were esterified to fatty acid methyl esters using two mL of 2% sodium hydroxide-methanol solution incubated in water bath at 85 °C for 30 min. Then three mL of 14% boron trifluoride-methanol solution was added, and the heating was continued at 85 ^∘^C for another 30 min for methylation. After the reaction, the mixture was cooled to room temperature. Subsequently, one mL of n-hexane was added to a centrifuge tube, and the mixture was vortexed for 2 min, then left to stand for 1 h to allow phase separation. The upper clear layer (100 μL) was taken and diluted to one mL with n-hexane. The solution was filtered through a 0.45 µm membrane filter. Finally, the fatty acid compositions were analyzed using a gas chromatograph (Trace 1310 ISQ, Thermo Fisher Scientific, Waltham, MA, USA) equipped with a fused capillary column (HP-88, 100 m × 0.25 mm × 0.20 µm, Thermo Fisher Scientific, Waltham, MA, USA).

### Statistical analysis

Data were presented as mean ± standard deviation (SD). Differences of RMR, AER and biochemical composition between two body parts after fission (anterior and posterior) and across six time points (10, 20, 30, 40, 50, and 60 d) along the regeneration period was analyzed using a two-way ANOVA with the GLM model in Rstudio-1.0.153. The normality of the data was assessed using the Shapiro–Wilk test, and the homogeneity of variances was examined using Levene’s test. The main factors in the model included two body parts after fission and six regeneration time points, along with their two-way interactions. Tukey’s multiple range test was conducted exclusively for significant interactions with package ‘emmeans’. Values of *p* < 0.05 was considered statistically significant. Linear regression was used to examine the relationship between time and body mass. A principal component analysis (PCA) was applied for the evaluation of fatty acids and amino acids composition. Before PCA analysis, percentage data were transformed following the equation: $tans{f}_{data}= \frac{180}{\pi } \times {\sin }^{-1}(\sqrt{ \frac{\mathrm{data}}{100} })$. The mean of the scores from PC-1 and PC-2 was calculated and plotted, and the differences were analyzed based on ANOVA.

## Results

### Routine metabolic rate, ammonium excretion and O:N ratio

**Figure 2:**
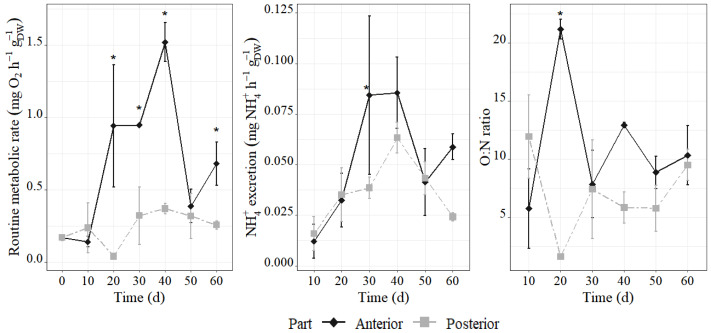
The metabolism and NH_4_^+^ excretion in anterior and posterior of *H. atra* during regeneration. (A) Routine metabolic rate (RMR, routine oxygen consumption rate, mg O_2_ h^−1^ g^−1^_DW_ (dryweight)). (B) The NH_4_^+^ excretion (AER, mg NH_4_^+^ h^−1^ g^−1^_DW_). (C) The O:N ratio (respiration/ ammonia excretion, by atoms). Values are means ± SD (*n* = 3, at each time point). An asterisk (*) indicates the significant difference between two fission part (two-way ANOVA simple effects, Tukey-adjusted *p* < 0.05).

The regeneration process is accompanied by significant change in routine metabolic rate (RMR) (Fig. 2A). RMR was significantly influenced over the time course of 60 days, with a significant difference between anterior and posterior during 20 to 40 and 60 days after fission. The RMR of whole individual before fission was around 0.17 ± 0.02 mg O_2_ h^−1^ g^−1^_DW_, while after fission, the anterior increased RMR from 0.64 ± 0.38 mg O_2_ h^−1^ g^−1^_DW_ 20 days after fission to 1.06 ± 0.46 mg O_2_ h^−1^ g^−1^_DW_ 40 days after fission (*p* < 0.05). Mean RMR in posterior fragments showed no significant difference throughout regeneration and averaged 0.25 ± 0.20 mg O_2_ h^−1^ g^−1^ DW. But the RMR slightly decreased to 0.04 ± 0.01 mg O_2_ h^−1^ g^−1^ DW by 20 days post-fission, before progressively increasing to a range of 0.325 ± 0.20 to 0.375 ± 0.05 mg O_2_ h^−1^ g^−1^ DW between 30- and 50-days post-fission.

Ammonium excretion rates also showed higher change at anterior than the posterior, similar trend with RMR (Fig. 2B). NH_4_^+^ excretion rate of the anterior increased from 0.012 ± 0.01 mg NH_4_^+^ h^−1^ g^−1^_DW_ 10 days after fission to 0.084–0.085 mg NH_4_^+^ h^−1^ g^−1^_DW_ 30–40 days after fission (*p* < 0.05). The posterior also showed progressively increased reaching to 0.063 ± 0.01 mg NH_4_^+^ h^−1^ g^−1^_DW_ during 30 to 40 days post-fission, but not significant differences over course of time. Accordingly, O:N ratio showed higher in the anterior than that of the posterior (Fig. 2C).

### Weight change and characteristics of fission part

**Figure 3:**
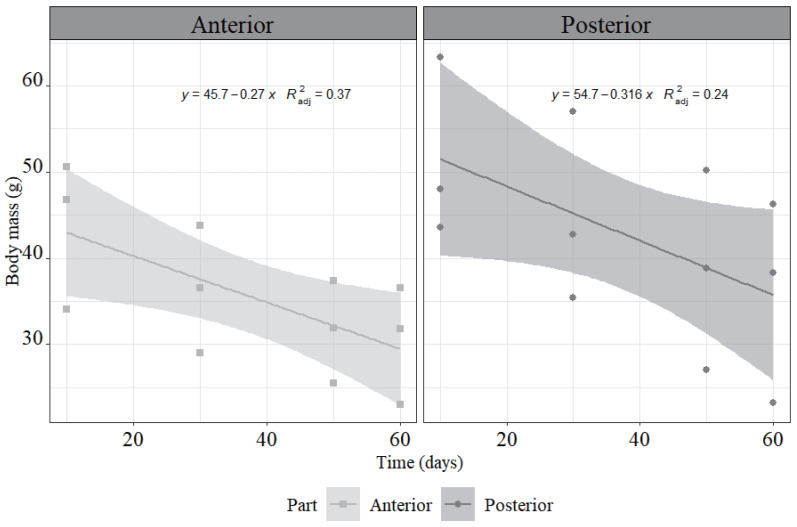
The body mass change of two fission parts during the regeneration time. Three individuals were split, and the body mass of the anterior and posterior was observed longitudinally over a 60-day regeneration period.

The weight of both anterior and posterior showed decreasing during the regeneration period (Fig. 3). Till 10 days post-fission, the total weight of the anterior and the posterior parts have decreased by approximately 31.64 ± 9.93% compared to the initial weight of the whole individuals. In the anterior segment, the mass decreased at an approximate rate of 0.27 g/d, reaching a final weight of 30.42 ± 6.87 g. In contrast, the posterior segment decreased at a rate of approximately 0.32 g/d, with a final weight of 35.92 ± 11.67 g.

**Figure 4:**
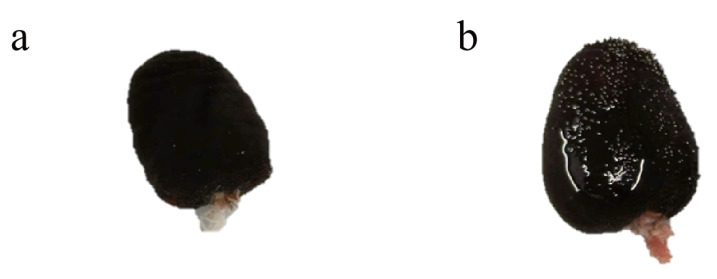
Rubber band-induced autotomy, and the two parts splits. (A) the anterior part with exposed intestines and respiratory trees (B) the posterior part with exposed respiratory trees.

Rubber band-induced autotomy into two parts in *H. atra* typically occurs within 5 to 15 h, exposing some internal organs (Fig. 4). The posterior part will absorb water immediately into the respiratory trees and expand till fission. After 24 to 48 h, the wound noticeably contracts, during which the sea cucumbers remain relatively immobile. By 72 h, some sea cucumbers, particularly the anterior, may expel most of their internal organs, causing the wound to reopen and increasing mortality. Around a week, the ruptured site generally shows signs of healing. Over day 10 to 30, the wound changed from a white surface to black tissue. By approximately 60 days, the anterior part develops an anus, and the posterior begins to grow head with tentacles. Survival rates varied over the course of the experiment. During the initial 10-day wound healing period, survival rates were low, with anterior and posterior segments exhibiting rates of approximately 45% and 55%, respectively. Thereafter, survival stabilized relatively, reaching 64–78% in both groups.

### Dry matter and ash content

The dry matter in the *H. atra* was average 13.4 ± 2.14% of the total weight at anterior, and 13.1 ± 1.59% at posterior, without any difference in the two parts (Table 1). The highest ash content appeared at peak at 10 days after fission, approximately 32% DW. The ash content decreased from 50 to 60 days during regeneration, with the ash level at 60 days post-fission being lower than that observed at 10-, 30-, and 40-days post-fission (*p* < 0.05). But there is slight change of ash content between anterior and posterior, with no significance.

**Table 1:** The dry matter and ash content during the regeneration period. Values are means ± SD. WW is wet weight, and DW is dry weight. Significant differences over regeneration time are indicated by different letters (*p* < 0.05).

Regeneration period (d)	Water content (%)		Dry matter content (% WW)		Ash content (% DW)	
	Anterior	Posterior	Anterior	Posterior	Anterior	Posterior
10	87.9 ± 0.6	86.9 ± 1.0	12.1 ± 1.0	13.1 ± 1.7	32.2 ± 1. 1^b^	31.8 ± 0.8^b^
20	85.2 ± 2.2	86.8 ± 0.7	14.8 ± 3.9	13.2 ± 1.2	29.3 ± 2.0^ab^	27.9 ± 1.49^ab^
30	87.1 ± 0.5	87.6 ± 0.3	12.9 ± 0.8	12.4 ± 0.5	31.8 ± 1.6^b^	30.5 ± 3.3^b^
40	85.8 ± 0.9	85.2 ± 1.5	14.3 ± 1.6	14.9 ± 2.6	31.8 ± 1.6^b^	30.6 ± 1.4^b^
50	86.3 ± 1.7	88.1 ± 0.4	13.7 ± 3.0	11.9 ± 0.7	25.3 ± 2.7^ab^	24.4 ± 0.6^ab^
60	87.5 ± 0.8	87.1 ± 0.8	12.5 ± 1.4	12.9 ± 1.5	19.9 ± 0.9^a^	21.5 ± 0.7^a^

### Biochemical composition

#### Protein, lipid and carbohydrate

**Figure 5:**
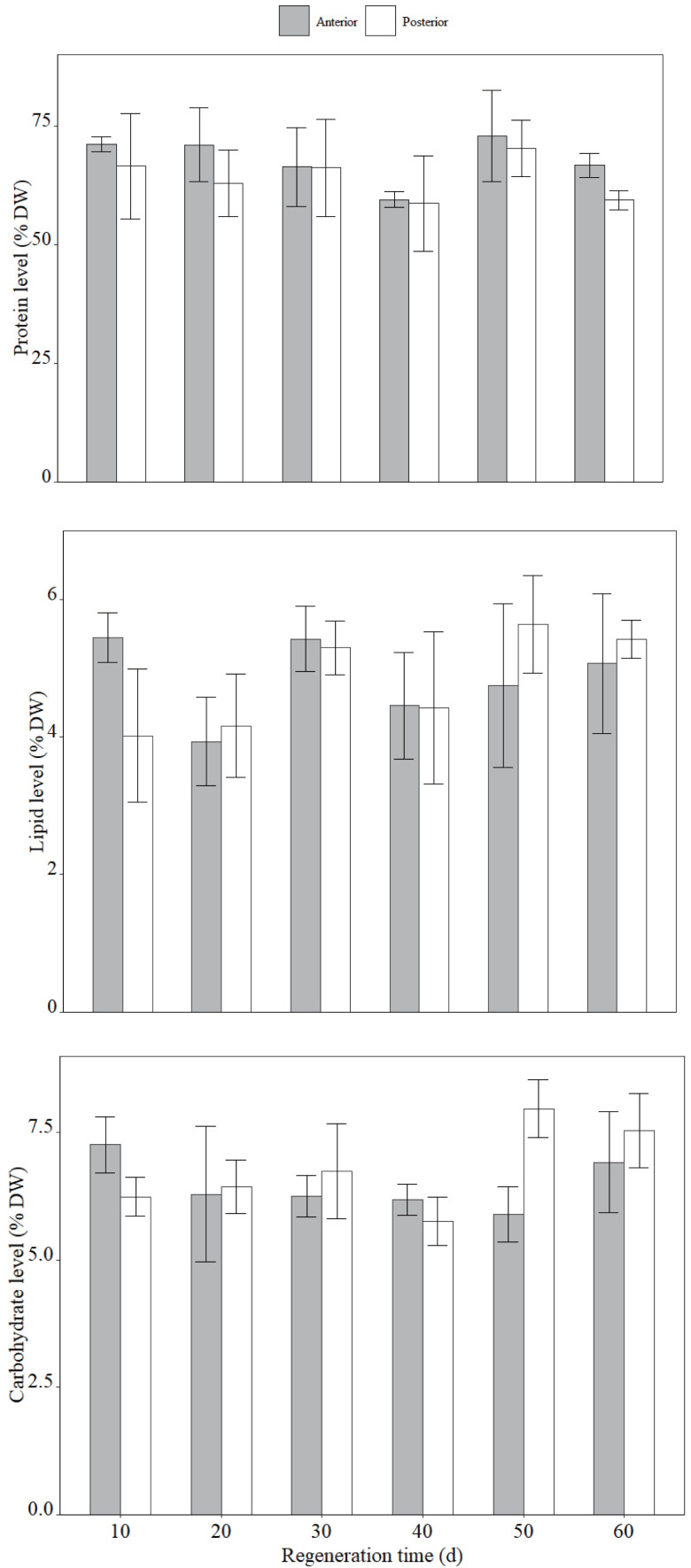
The main biochemical components of two fission parts from *H.atra* during the regeneration. (A) Protein level (% DW), (B) Lipid level (% DW), (C) Carbohydrate level (% DW).

The protein, lipid and carbohydrate of *H. atra* remained largely unchanged during regeneration (Fig. 5). Both protein and lipid contents showed no significant variation at two fission part over the regeneration course. Protein levels were 67.9 ±.9% DW in the anterior region and 63.9 ± 8.2% DW in the posterior. But the protein in both anterior and posterior was found to be decreased during the 30th -40th regeneration. Similarly, lipid content was constant, with 4.85 ± 0.87% DW in the anterior and 4.83 ± 0.92% DW in the posterior. No significant change in carbohydrate content was observed in the anterior during regeneration. In contrast, the posterior exhibited higher carbohydrate levels between 50 and 60 days after fission compared to 40 days after fission (*p* < 0.05). In addition, on 50 days after fission, the posterior had higher carbohydrate than the anterior (*p* < 0.05).

#### Amino acids and fatty acids profile

**Figure 6:**
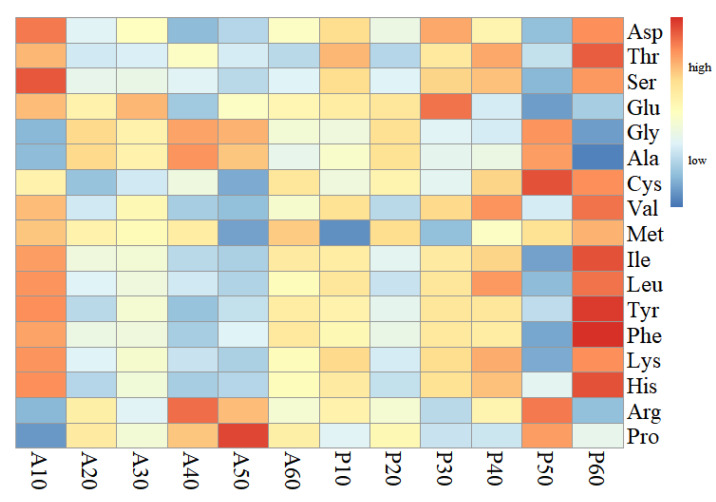
Heatmap of amino acids by hierarchical clustering analysis under different treatments. A10-A60 refers to the anterior part during the 10, 20, 30, 40, 50, and 60 regenerations, while P10-P60 refers to the posterior during the same regeneration period. Data was arcsine transformed. Asp: aspartic acid/asparagine, Glu: glutamic acid/glutamine, Ser: serine, Gly: glycine, His: histidine, Arg: arginine, Thr: threonine, Ala: alanine, Pro: proline, Tyr: tyrosine. Val: valine, Met: methionine Ile: isoleucine, Leu: leucine, Phe: phenylalanine, Lys: lysine.

The amino acids profile in the anterior and posterior parts of *H. atra* over the regeneration course was shown in Table S1 and Fig. S1. The AA composition, the factor loadings and the heatmap indicated minor difference in two planes during the regeneration time (Fig. 6).

The fatty acid profile of two fission planes of the sea cucumber *H. atra* during the regeneration process is presented in Table S2. The content of fatty acids C20:4 n-6, C18:1 n-9, C22:1 n-9, C16:0 and C24:1 were the predominant components among all fatty acids. Saturated fatty acids C14:0, C15:0 and C17:0 in the anterior part 10 days after fission was significantly higher compared to the posterior part, while the opposite was observed 40 days after fission (*p* < 0.05). C20:5n-3 in the posterior part showed significantly higher than that in the anterior part during 10 to 30 days after fission, while lower during 40 and 60 days (*p* < 0.05). The total fatty acids, SFA, MUFA, C14:0, C15:0, C17:0, C18:0, C18:1n-9 and C18:2n-6 increased significantly 30 days after fission both at the anterior and posterior (*p* < 0.05).

**Figure 7:**
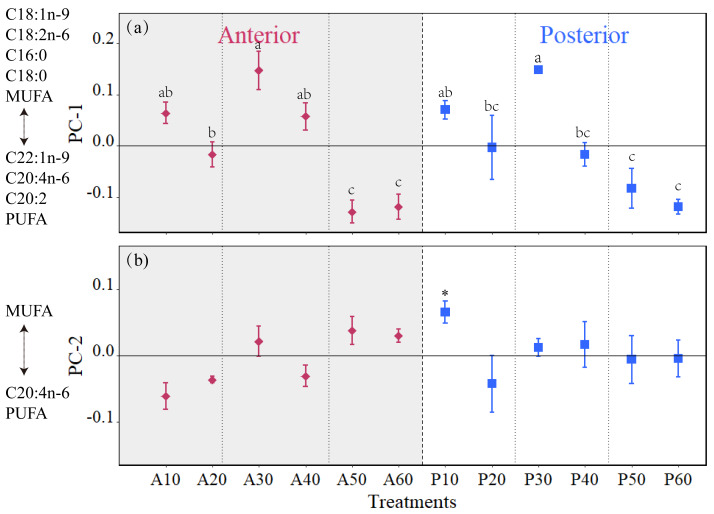
The characteristics of the anterior and posterior on the first (PC-1; a) and second (PC-2; b) principal components generated from a principal components analysis (PCA) on *H. atra* fatty acids. Data were arcsine transformed. The values in y-coordinate are the mean of the loadings from PC-1 and PC-2. PC-1 and PC-2 accounted for 70 and 16% of the variation in the data set, respectively. The first ten contributors are listed in Table S3. For PC-1, fatty acids C18:1n-9, C18:2n-6, C16:0, C18:0, and MUFA loaded positively, while C22:1n-9, C20:4n-6, C20:2, and PUFA loaded negatively. For PC-2, 20:4n-6 and PUFA loaded negatively. SFA: saturated fatty acids; MUFA: monounsaturated fatty acids; PUFA: polyunsaturated fatty acids. The lowercase letters indicated significant difference over the regeneration time in the same segmented part (*p* < 0.05). The asterisk “*” mean the significant difference between the anterior and the posterior at the same time (*p* < 0.05). A10-A60 refers to the anterior part during the 10, 20, 30, 40, 50, and 60 regenerations, while P10-P60 refers to the posterior during the same regeneration period.

PCA analysis revealed that the fatty acid composition in *H. atra* is significantly influenced by both fission plates and the regeneration time (Fig. 7). PC-1 explained 70% of the variance. Fatty acids of C18:1 n-9, MUFA, C16:0, C18:2 n-6, C18:0, C22:1 n-9, PUFA, C20:2 and C20:4 n-6 were dominantly different among the groups. PC-1 were associated with relatively higher level of C18:1 n-9, C18:2 n-6, C16:0, C16:0 and MUFA, relatively lower level of C22:1 n-9, C20:4 n-6, C20:2 and PUFA. PC-2 accounted for 16% of the variation. Positive loadings on PC-2 were also associated with higher MUFA. Based on ANOVA using the scores from PC-1 and PC-2, there were no significant difference between the two fission parts at the same regeneration time. However, significant differences were observed over the regeneration periods at 30, 50 and 60 days both in anterior and posterior parts.

## Discussion

In this study, *H. atra* successfully regenerated both their anterior regions and posterior parts following induced fission at the mid-body. Consistent with previous reports (Dolmatov, 2014a; Dolmatov, 2014b), the anterior-to-posterior length ratio was approximately 4:5, with the posterior fragment slightly larger. This asymmetric division may have adaptive significance, as the posterior fragment retains the cloaca and a larger portion of the digestive tract, potentially enhancing post-fission survival.

A slight difference in regeneration rate was noted between the anterior and posterior fragments. In surviving individuals, intestinal reconnection along the pre-existing side was completed in both fragments by day 7. The respiratory trees remained largely intact in the posterior fragment. The anterior fragment achieved a complete but smaller transparent respiratory tree by day 50 and reached full development within 50-60 days. In contrast, regeneration of the head, including tentacles, in the posterior fragment required 60 days or more. This study found ejecting the viscera in response to fission stress was generally fatal. Interestingly, a previous study on fission of sea cucumber *H. polii* showed higher survival rate in evisceration induction before fission induction than the non-eviscerated group (Moussa, Nasef & Abdel-Latif, 2019). But we found the sea cucumber can use the left organs to develop, which may allow the regenerating fragments to reduce the costly and time-intensive process of *de novo* organ formation.

**Figure 8:**
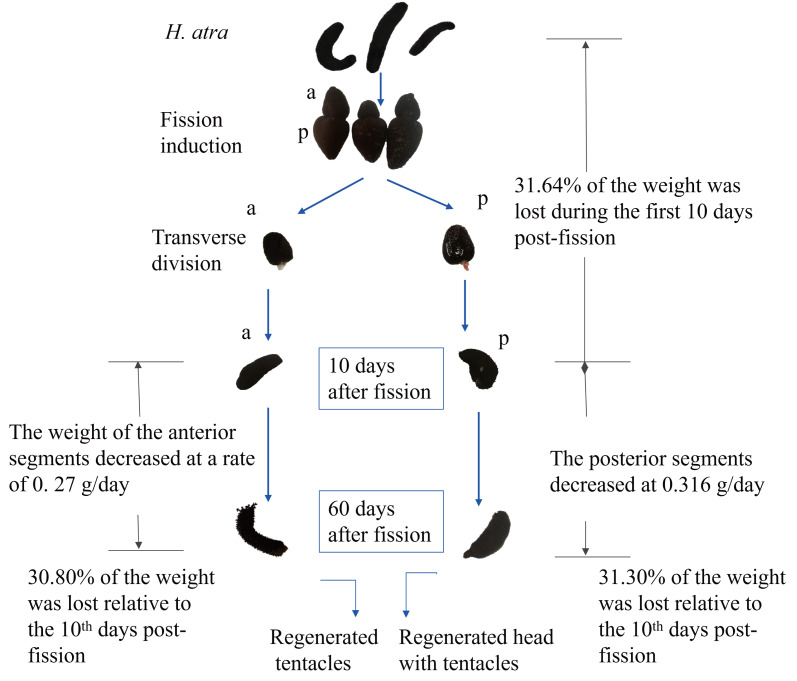
Schematic diagram of the body weight change in *H. atra* during regeneration.

After fission, the anterior fragment contained the aquapharyngeal complex, and a portion of the gut without a cloaca, and the respiratory trees. Whereas the posterior fragment retained the cloaca, a large gut section, and respiratory trees, but lacked the aquapharyngeal complex and tentacles (Asha & Diwakar, 2015; Dolmatov, 2014a; Dolmatov, 2014b; Dolmatov, Khang & Kamenev, 2012; Wilkie, 2001). Therefore, unlike stellate echinoderms (such as crinoids, ophiuroids, and asteroids) that can continue feeding after arm loss, fission in sea cucumbers results in the complete loss of a functional digestive system in both fragments, precluding feeding until regeneration is complete (Dolmatov, 2014a; Dolmatov, 2014b; Lawrence, 2010). The energetic cost of asexual fission in sea cucumbers includes both reduced ability to obtain food and energy allocation for regenerating lost body parts (Lawrence, 2010). In this study, we speculate *H. atra* responded to starvation during asexual reproduction by decreasing body mass (Fig. 8), as the reduction in body mass is known as the most evident and frequently documented response to starvation (Marshall, 2010).

Ash content showed a progressive decline starting from day 50, with a statistically significant decrease observed at day 60 compared to other time points. The reduction in ash content at later stages likely reflects the progressive atrophy of body wall tissues. The significant decrease in ash content during the late starvation period may indicate that the sea cucumber began to resorb its calcareous ossicles. These structures, whose precipitation is induced by organic compounds such as glycoproteins (Dubois, 1990), are likely as a strategy to maintain ionic homeostasis and basic physiological functions along with the consumption of organic energy. Further research is needed to confirm this proposed mechanism.

Furthermore, routine metabolic rate (RMR) was significantly higher in the anterior fragment than in the posterior fragment, particularly between 20- and 40-days post-fission, which means higher metabolic rate in the anterior part during this period. Previous studies have shown that lower temperature or salinity fluctuation reduces RMR in *H. atra*, whereas exposure to 30 °C increases RMR to approximately 1.15 mg O_2_ h^−^^1^ g^−^^1^ DW (AbouElmaaty et al., 2023). In this study, the highest RMR (1.06 ± 0.46 mg O_2_ h^−^^1^ g^−^^1^ DW) was observed in the anterior fragment at 40 days post-fission. This value was comparable to that induced by thermal stress, which indicates that the elevated metabolic rate supports the energetic requirements of regeneration. In contrast, the RMR in the posterior fragment remained stable during the regeneration, which means the posterior may require lower energetic investment than the anterior for regeneration. Meanwhile, ammonia excretion rate increased in both fragments after 20 days post-fission, with higher rates in the anterior part. Increased ammonia excretion typically indicates protein catabolism (Chew et al., 2005). The O:N ratio in the anterior fragment at 20 days was approximately 20, indicating that a mixed fuel source of lipids and proteins. At most other times, ratio below 10 suggested that protein served as the primary energy substrate (Yang et al., 2006).

Study showed that cells obtained from primary intestinal culture 14 to 16 days post evisceration were actively proliferating due to a specific state of the epithelia involved in regeneration. In regenerating organisms, the level of DNA synthesis exceeded the normal level 3 to 4 times, even on the 20th day of regeneration (Odintsova, Dolmatov & Mashanov, 2005). Therefore, the increased RMR and nitrogen excretion rate observed in the present study may reflect the energetic demands of wound healing and tissue remodeling.

During regeneration, the typical paradigm of energy substrate utilization, carbohydrates first, followed by lipids, and finally protein (Feral, 1985; Marshall, 2010). But it was not supported by our proximate composition analysis. In this study, no significant changes were detected in the protein, lipid, and carbohydrate content in tissues of *H. atra* throughout the regeneration period. Yang et al. (2022) found that long-term starvation (four months) has minimal impact on primary metabolite concentrations in crown-of-thorns starfish, with gene expression analysis indicating a hypometabolic state as an adaptive response. Therefore, this stability in the proximate components of sea cucumbers may be attributed to their status as low-energy organisms.

The body wall of holothurians serves as a significant storage depot, primarily for proteins (Feral, 1985; Kühnhold et al., 2017), with protein content in *H. atra* reaching approximately 65.92 g/100 g. This reservoir may buffer fluctuations in whole-body composition during fasting. Moreover, sea cucumber may utilize alternative nutrient sources during regeneration. Feral (1985) demonstrated trans-integumentary uptake of dissolved organic matter in *Leptosynapta galliennei*, potentially mediated by subcuticular bacteria. Similarly, sea cucumber *Parastichopus californicus* assimilates free amino acids *via* its respiratory trees to regenerate its internal organs (Brothers, Lee & Nestler, 2015). Whether *H. atra* employs such mechanisms during fission-induced regeneration warrants further investigation.

Although proximate composition remained stable, fatty acids profiles exhibited pronounced temporal dynamics during regeneration. The fatty acid composition in tissue of animal can be affected by nutritional stress and starvation (Marshall, 2010). During the asexual regeneration, the saturated fatty acids decreased compared with the integrated individuals. The relative proportions of fatty acids C18:1n-9, C18:2n-6, C16:0, C18:0, and monounsaturated fatty acids (MUFA) were elevated at day 30 before declining between days 50 and 60. In contrast, C20:4n-6, C20:2, and total polyunsaturated fatty acids (PUFA) showed an inverse trend. Total fatty acids content was significantly increased at day 30 in both anterior and posterior part the show significantly higher than the other time. On day 30, total fatty acid content in both anterior and posterior fragments, indicating a peak in lipid metabolic activity during mid-regeneration.

This transient elevation likely reflect increased membrane synthesis, energy demand, and cellular signaling associated with tissue remodeling (Monroig, Tocher & Navarro, 2013). Fatty acids play crucial roles not only in energy storage but also in maintaining membrane integrity and mediating signaling pathways during regeneration (Yaqoob & Calder, 2007). The observed temporal shifts in fatty acid profiles likely correspond to distinct metabolic requirements during early wound healing, tissue proliferation, and late differentiation stages.

In natural habitats, *H. atra* often exhibits high-density patchy distribution linked to substrate organic content (Pierrat et al., 2024). Fission has been observed primarily during colder seasons when populations are dense, suggesting that food scarcity and lower temperatures may trigger autotomy (Asha & Diwakar, 2015; Lawrence et al., 1986). The regeneration process following injury is similar for both the anterior and posterior fragments, with minimal energy expenditure. This timing suggests that regeneration may be a response to both food scarcity and low temperatures.

In conclusion, this study demonstrates that *H. atra* can successfully regenerate both anterior and posterior structures following transverse division, despite the complete loss of a functional digestive system. During the asexual regeneration period, the sea cucumber exhibits fragment-specific metabolic responses, with higher RMR and ammonia excretion in the anterior fragment. Significant fatty acid dynamics despite stable proximate composition, reflecting a complex energy allocation strategy. A potential hypometabolic state that minimizes energy expenditure while prioritizing regeneration. These findings provide new insights into the physiological strategies that enable asexual reproduction in tropical sea cucumbers and have implications for understanding population dynamics and conservation of these ecologically important species.

## Supplemental Information

10.7717/peerj.21358/supp-1 Supplemental Information 1 Supplemental figures and tables

10.7717/peerj.21358/supp-2 Supplemental Information 2 The data to analyse the amino acids

10.7717/peerj.21358/supp-3 Supplemental Information 3 Data to calculate the oxygen consumption and amonia excretion rate

10.7717/peerj.21358/supp-4 Supplemental Information 4 Data for Figure 7

10.7717/peerj.21358/supp-5 Supplemental Information 5 Data to calculate the main biochemical composition

## Additional Information and Declarations

### Data Availability

The following information was supplied regarding data availability: The raw measurements are available in the Supplemental Files.
