# Supplementary material for: Individual response in basal metabolism and nutritional composition to asexual reproduction in tropical sea cucumber Holothuria atra
Source: PeerJ. 2026 Jun 2;14:e21358. doi: 10.7717/peerj.21358 (PMC13239475; doi:10.7717/peerj.21358)
Supplement: Supplemental Information 1 [file peerj-14-21358-s001.docx]

**Supplement**

**Table S1** Amino acids profile at anterior and posterior *H. atra* during the regeneration after fission. 10, 20 30, 40, 50, 60 were days after fission. Asterisk’*’denote the significant difference between two fission part. Different superscript letters within rows denoted significant differences over the regeneration time (p < 0.05) in descending order. Data were presented as mean ± SE (n = 3). Asp: aspartic acid/asparagine, Glu: glutamic acid/glutamine, Ser: serine, Gly: glycine, His: histidine, Arg: arginine, Thr: threonine, Ala: alanine, Pro: proline, Tyr: tyrosine. Val: valine, Met: methionine Ile: isoleucine, Leu: leucine, Phe: phenylalanine, Lys: lysine. TAA: total amino acids.

| % TAA |  |  |  | Anterior |  |  |  |
| --- | --- | --- | --- | --- | --- | --- | --- |
|  | Initial | 10 | 20 | 30 | 40 | 50 | 60 |
| Asp | 11.25±1.08 | 10.41±0.15 | 10.03±0.33 | 10.14±0.4 | 9.90±0.19 | 9.96±0.31 | 10.12±0.35 |
| Thr | 5.46±0.73 | 5.03±0.12 | 4.91±0.11 | 4.91±0.06 | 4.96±0.07 | 4.91±0.11 | 4.90±0.12 |
| Ser | 4.12±0.17 | 4.12±0.09 | 3.86±0.17 | 3.87±0.26 | 3.85±0.14 | 3.81±0.10 | 3.85±0.18 |
| Glu | 13.65±9.43 | 20.07±0.94 | 19.77±1.71 | 20.08±1.14 | 19.19±0.71 | 19.65±0.74 | 19.75±1.20 |
| Gly | 14.22±1.36 | 15.43±0.30 | 17.47±1.55 | 17.02±2.30 | 17.9±1.05 | 17.79±0.82 | 16.51±2.02 |
| Ala | 8.40±1.41 | 8.12±0.11 | 8.79±0.57 | 8.65±0.71 | 8.99±0.35 | 8.85±0.31 | 8.39±0.70 |
| Cys | 0.48±0.13 | 0.40±0.09 | 0.19±0.12 | 0.25±0.04 | 0.31±0.08 | 0.16±0.04 | 0.45±0.21 |
| Val | 4.35±0.50 | 3.69±0.17 | 3.44±0.12 | 3.57±0.27 | 3.38±0.11 | 3.36±0.17 | 3.53±0.24 |
| Met | 0.72±0.17 | 0.96±0.14 | 0.84±0.04 | 0.80±0.07 | 0.86±0.14 | 0.51±0.44 | 0.96±0.10 |
| ILe | 3.30±0.40 | 2.66±0.06 | 2.39±0.07 | 2.41±0.33 | 2.29±0.16 | 2.27±0.10 | 2.53±0.26 |
| Leu | 5.11±0.55 | 4.33±0.13 | 3.82±0.16 | 3.90±0.51 | 3.78±0.25 | 3.71±0.16 | 4.00±0.44 |
| Tyr | 3.07±0.57 | 2.43±0.05 | 2.16±0.20 | 2.25±0.33 | 2.13±0.16 | 2.17±0.11 | 2.32±0.20 |
| Phe | 3.46±0.51 | 2.46±0.05 | 2.17±0.14 | 2.19±0.36 | 2.05±0.14 | 2.13±0.17 | 2.33±0.31 |
| Lys | 4.31±1.02 | 3.33±0.12 | 2.7±0.36 | 2.86±0.74 | 2.63±0.32 | 2.57±0.19 | 2.92±0.61 |
| His | 1.50±0.28 | 1.10±0.05 | 0.88±0.10 | 0.95±0.24 | 0.88±0.09 | 0.88±0.09 | 0.98±0.18 |
| Arg | 8.44±1.61 | 7.58±0.14 | 7.81±0.40 | 7.68±0.32 | 7.97±0.17 | 7.89±0.23 | 7.73±0.36 |
| Pro | 8.16±1.09 | 7.87±0.10 | 8.77±0.78 | 8.48±1.02 | 8.95±0.59 | 9.39±0.39 | 8.74±0.85 |
| TAA  (g/100g Protein) | 5.78±0.74 | 7.73±1.13 | 9.64±1.91 | 8.62±1.75 | 12.34±1.78 | 9.03±2.98 | 9.30±0.11 |

|  |  |  | | Posterior |  |  |  |
| --- | --- | --- | --- | --- | --- | --- | --- |
| % TAA | Initial | 10 | 20 | 30 | 40 | 50 | 60 |
| Asp | 11.25±1.08 | 10.26±0.4 | 10.06±0.09 | 10.33±0.22 | 10.18±0.25 | 9.90±0.17 | 10.38±0.47 |
| Thr | 5.46±0.73 | 5.03±0.08 | 4.89±0.18 | 4.99±0.06 | 5.04±0.06 | 4.90±0.22 | 5.07±0.21 |
| Ser | 4.12±0.17 | 4.00±0.15 | 3.85±0.19 | 4.01±0.12 | 4.03±0.25 | 3.76±0.12 | 4.06±0.32 |
| Glu | 13.65±9.43 | 19.84±1.69 | 19.88±0.83 | 20.31±0.62 | 19.37±0.46 | 19.00±1.51 | 19.20±0.92 |
| Gly | 14.22±1.36 | 16.45±2.40 | 17.41±0.54 | 16.16±1.06 | 16.07±2.78 | 18.05±0.43 | 15.22±3.30 |
| Ala | 8.40±1.41 | 8.51±0.82 | 8.75±0.17 | 8.39±0.36 | 8.43±0.88 | 8.96±0.16 | 7.95±1.07 |
| Cys | 0.48±0.13 | 0.31±0.09 | 0.40±0.08 | 0.28±0.11 | 0.49±0.28 | 0.69±0.04 | 0.60±0.41 |
| Val | 4.35±0.50 | 3.64±0.21 | 3.41±0.19 | 3.66±0.18 | 3.74±0.37 | 3.44±0.30 | 3.78±0.45 |
| Met | 0.72±0.17 | 0.48±0.54 | 0.92±0.01 | 0.55±0.50 | 0.77±0.67 | 0.90±0.07 | 1.00±0.27 |
| ILe | 3.30±0.40 | 2.52±0.28 | 2.35±0.12 | 2.53±0.12 | 2.58±0.47 | 2.19±0.14 | 2.77±0.44 |
| Leu | 5.11±0.55 | 4.12±0.45 | 3.76±0.26 | 4.13±0.21 | 4.32±0.75 | 3.63±0.27 | 4.39±0.85 |
| Tyr | 3.07±0.57 | 2.30±0.21 | 2.21±0.15 | 2.33±0.15 | 2.34±0.25 | 2.16±0.08 | 2.51±0.38 |
| Phe | 3.46±0.51 | 2.27±0.30 | 2.17±0.12 | 2.33±0.08 | 2.32±0.41 | 1.98±0.16 | 2.61±0.58 |
| Lys | 4.31±1.02 | 3.15±0.69 | 2.67±0.27 | 3.13±0.30 | 3.26±0.92 | 2.44±0.19 | 3.35±1.05 |
| His | 1.50±0.28 | 1.01±0.22 | 0.89±0.05 | 1.03±0.10 | 1.06±0.25 | 0.92±0.20 | 1.14±0.29 |
| Arg | 8.44±1.61 | 7.80±0.47 | 7.74±0.09 | 7.63±0.20 | 7.79±0.27 | 7.96±0.17 | 7.60±0.34 |
| Pro | 8.16±1.09 | 8.32±0.87 | 8.64±0.28 | 8.21±0.53 | 8.22±1.14 | 9.10±0.43 | 8.37±1.19 |
| TAA  (g/100g Protein) | 5.78±0.74 | 8.85±2.34 | 9.86±2.07 | 9.25±1.70 | 10.80±0.26 | 8.95±1.02 | 10.08±1.09 |

Values expressed as mean ± standard deviation. According to ANOVA, different lowercase letters indicate significant mean differences among regeneration period, followed by posthoc Tukey test, at 5% probability. Asterisk (*) represents the difference between two body parts (Anterior vs Posterior) in relation to the different regeneration period. Different letters mean significant different values over the time.


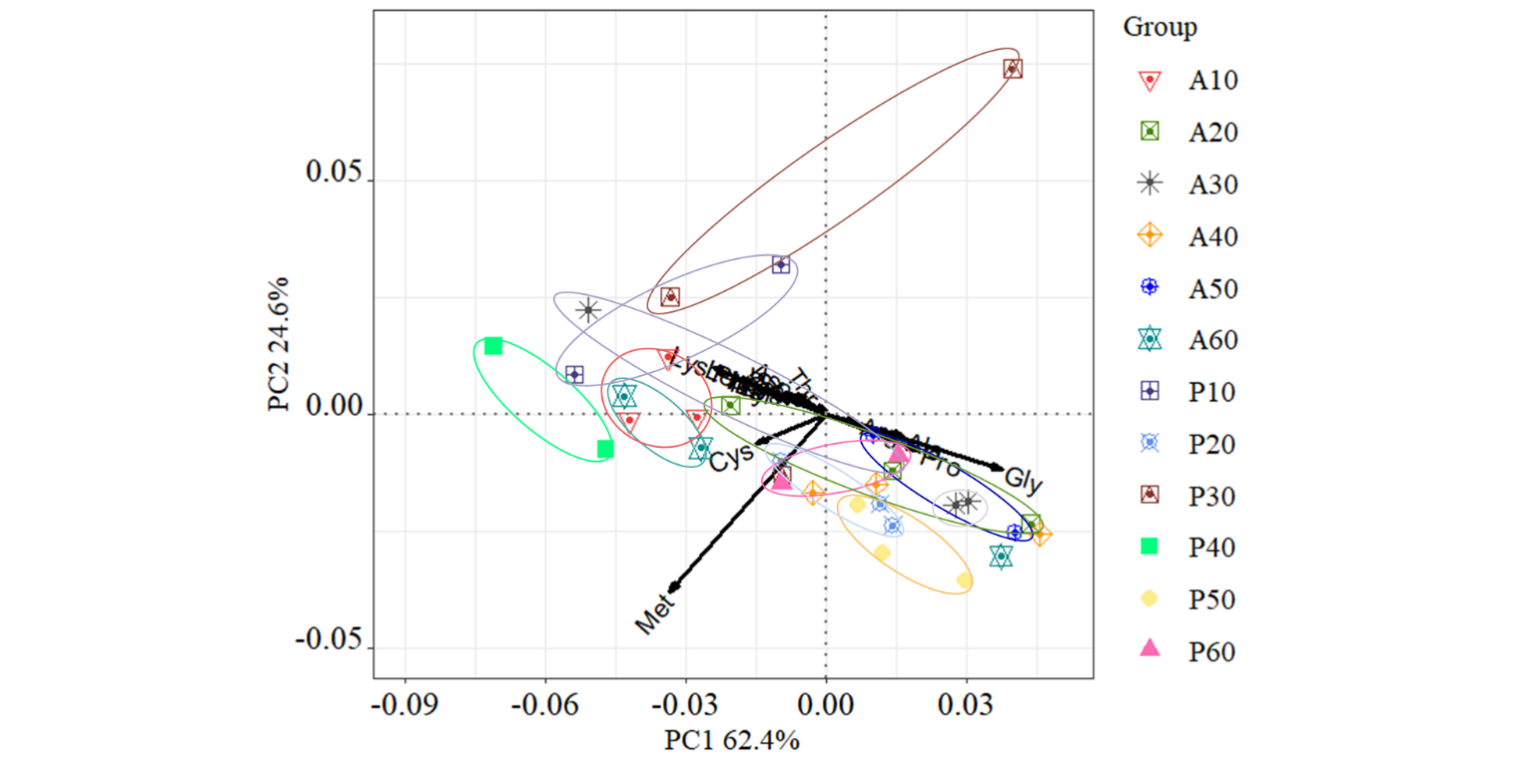


**Fig. S1** Principal component analysis of amino acids (% of the total amino acids) in two fission planes of sea cucumber *H. atra* over the regeneration course. A10-A60 refers to the anterior part during the 10, 20, 30, 40, 50, and 60 regenerations, while P10-P60 refers to the posterior during the same regeneration period. Data was arcsine transformed. Asp: aspartic acid/asparagine, Glu: glutamic acid/glutamine, Ser: serine, Gly: glycine, His: histidine, Arg: arginine, Thr: threonine, Ala: alanine, Pro: proline, Tyr: tyrosine. Val: valine, Met: methionine Ile: isoleucine, Leu: leucine, Phe: phenylalanine, Lys: lysine.

**Table S2** Fatty acids profile at anterior and posterior of *H. atra* during the regeneration after fission. 10, 20 30, 40, 50 and 60 were days after fission. Data were presented as mean ± SE (n = 3). Different superscript letters within rows denoted significant differences over the regeneration time (p < 0.05) in descending order with the Initial. Asterisk’*’denote the significant difference between two fission parts.

| %TFA |  |  |  | Anterior |  |  |  |
| --- | --- | --- | --- | --- | --- | --- | --- |
|  | Initial | 10 | 20 | 30 | 40 | 50 | 60 |
| C14:0 | 0.69±0.01 | 0.59±0.11 | 0.70±0.06 | 0.95±0.24 | 1.12±0.1 | 0.80±0.33 | 0.87±0.22 |
| C15:0 | 0.19±0.01 | 0.07±0.01* | 0.08±0.03 | 0.10±0.02 | 0.14±0.05* | 0.10±0.03 | 0.13±0.04 |
| C16:0 | 11.43±0.04 | 9.46±0.91^ab^ | 7.98±0.62^ab^ | 12.51±2.44^c^ | 9.84±1.12^b^ | 6.30±1.21^a^ | 7.55±1.12^ab^ |
| C17:0 | 0.52±0.01 | 0.22±0.03^a*^ | 0.37±0.07^ab^ | 0.43±0.05^b*^ | 0.44±0.06^b*^ | 0.34±0.12^ab^ | 0.40±0.03^b^ |
| C18:0 | 9.25±0.04 | 7.47±0.35^a^ | 7.27±0.28^a^ | 9.02±0.48^b^ | 8.12±0.49^b^ | 6.39±0.39^a^ | 6.62±0.29^a^ |
| C20:0 | 4.23±0.02 | 4.24±0.16^b*^ | 4.33±0.09^b^ | 2.88±0.39^a*^ | 3.72±0.30^b^ | 4.42±0.09^b^ | 4.24±0.22^b^ |
| C21:0 | 4.57±0.12 | 5.29±0.02* | 5.71±0.24 | 4.62±0.43 | 5.06±0.34 | 5.65±0.30 | 5.61±0.55 |
| C22:0 | 6.01±0.06 | 6.20±0.38^ab^ | 7.03±0.27^b^ | 5.64±1.15^a^ | 6.11±0.37^ab^ | 6.86±0.07^ab^ | 6.82±0.45^ab^ |
| C23:0 | 2.63±0.06 | 3.10±0.24 | 3.50±0.22 | 2.80±0.56 | 3.03±0.22 | 3.34±0.16 | 3.47±0.28 |
| C24:0 | 1.12±0.02 | 1.28±0.27 | 1.30±0.08 | 1.07±0.22 | 1.10±0.15 | 1.25±0.05 | 1.29±0.03 |
| C15:1n-5 | / | 0.09±0.01 | 0.08±0.01 | 0.07±0.02 | 0.07±0.01 | 0.07±0.02* | 0.10±0.03 |
| C16:1n-7 | 1.67±0.03 | 0.97±0.10^ab^ | 0.85±0.09^ab^ | 0.98±0.15^ab^ | 1.32±0.3^b*^ | 0.76±0.24^a^ | 0.90±0.34^ab^ |
| C18:1n-9c | 13.9±0.03 | 13.6±1.72^bc^ | 9.71±2.04^b^ | 15.6±2.59^c^ | 12.1±1.69^bc^ | 5.99±0.78^a^ | 5.93±1.34^a^ |
| C20:1n-9 | 1.67±0.02 | 1.68±0.06^ab*^ | 1.58±0.14^a^ | 1.48±0.09^a^ | 1.98±0.11^b^ | 1.84±0.29^ab^ | 1.65±0.06^ab^ |
| C22:1n-9 | 1.14±0.16 | 8.23±1.41^a*^ | 10.9±0.84^ab^ | 10.28±1.3^ab^ | 9.27±2.3^ab*^ | 16.6±2.57^c^ | 15.5±0.38^bc^ |
| C24:1 | 8.44±0.33 | 9.74±0.98^ab^ | 10.67±1.13^b^ | 8.53±1.23^a^ | 9.98±0.82^ab^ | 9.92±0.48^ab^ | 10.39±0.45^ab^ |
| C18:2n-6c | 1.83±0.02 | 2.26±0.47^bc^ | 1.47±0.54^ab^ | 2.88±0.75^c^ | 1.85±0.37^bc^ | 0.81±0.60^a^ | 0.43±0.16^a^ |
| C20:2 | / | 0.75±0.18^a^ | 0.86±0.04^a^ | 0.56±0.09^a^ | 0.69±0.09^a^ | 2.31±0.28^b*^ | 2.20±0.26^b^ |
| C20:4n-6 | 17.21±0.08 | 21.89±1.7^ab*^ | 22.29±0.4^b^ | 17.51±2.2^a^ | 20.00±0.8^ab^ | 22.42±0.5^b^ | 22.16±1.5^ab^ |
| C20:3n-6 | 0.26±0.03 | / | / | / | / | / | / |
| C20:3n-3 | 1.26±0.08 | / | / | / | / | / | / |
| C22:2 | / | 0.19±0.03^ab^ | 0.18±0.02^ab^ | 0.13±0.02^a^ | 0.14±0.03^a^ | 0.25±0.06^b*^ | 0.19±0.01^ab^ |
| C20:5n-3 | 11.74±0.02 | 2.70±0.15^ab^ | 3.13±0.66^ab^ | 1.93±0.50^a^ | 3.93±0.44^b^ | 3.60±0.65^b^ | 3.54±0.47^b^ |
| C22:6n-3 | 0.26±0.05 | / | / | / | / | / | / |
| SFA | 45.38±0.13 | 37.90±0.90^abc^ | 38.30±0.60^abc^ | 40.00±1.80^c^ | 38.7±1.10^bc*^ | 35.5±2.0^a^ | 37.0±0.3^ab^ |
| MUFA | 30.44±0.23 | 34.31±1.11 | 33.79±0.12 | 36.98±2.37 | 34.71±0.39 | 35.16±2.54 | 34.47±1.05 |
| PUFA | 24.18±0.31 | 27.79±1.88^b*^ | 27.94±0.47^b^ | 23.01±1.52^a^ | 26.6±0.79^ab^ | 29.38±0.71^b^ | 28.52±1.14^b^ |
| TFA (mg/100gDW) | 110.85±8.97 | 123.8±6.00^a^ | 134±10.3^ab^ | 156.3±10.3^b^ | 121.3±9.9^a^ | 112.5±11.0^a^ | 130.4±5.0^a^* |

| % TFA |  |  | | Posterior |  |  |  |
| --- | --- | --- | --- | --- | --- | --- | --- |
|  | Initial | 10 | 20 | 30 | 40 | 50 | 60 |
| C14:0 | 0.69±0.01 | 0.85±0.11 | 0.64±0.09 | 0.82±0.19 | 0.66±0.03 | 0.71±0.17 | 0.82±0.07 |
| C15:0 | 0.19±0.01 | 0.15±0.04* | 0.08±0.02 | 0.10±0.01 | 0.07±0.01* | 0.12±0.04 | 0.10±0.01 |
| C16:0 | 11.43±0.04 | 10.44±1.05 | 7.97±2.50 | 12.15±0.16 | 7.45±0.55* | 7.02±0.98 | 7.76±0.51 |
| C17:0 | 0.52±0.01 | 0.36±0.05 | 0.35±0.02 | 0.40±0.03 | 0.28±0.04* | 0.37±0.12 | 0.37±0.05 |
| C18:0 | 9.25±0.04 | 8.32±0.48^bc^ | 7.35±0.89^ab^ | 9.22±0.21^c^ | 6.87±0.42^a^ | 6.74±0.19^a^ | 7.07±0.15^a^ |
| C20:0 | 4.23±0.02 | 3.65±0.18^ab*^ | 4.35±0.43bc | 3.40±0.14^a*^ | 4.18±0.32^bc^ | 4.19±0.49^bc^ | 4.39±0.23^c^ |
| C21:0 | 4.57±0.12 | 3.71±0.69^a*^ | 5.01±1.11^ab^ | 4.10±0.27^ab^ | 5.40±0.31^b^ | 5.28±0.72^b^ | 5.23±0.37^2^ |
| C22:0 | 6.01±0.06 | 5.70±0.18^ab^ | 6.84±1.02^b^ | 5.43±0.27^a^ | 6.67±0.05^ab^ | 6.96±0.33^b^ | 6.87±0.24^b^ |
| C23:0 | 2.63±0.06 | 2.59±0.11^a^ | 3.35±0.55^ab^ | 2.66±0.27^a^ | 3.30±0.16^ab^ | 3.63±0.37^b^ | 3.58±0.22^n-5^ |
| C24:0 | 1.12±0.02 | 1.36±0.42 | 1.39±0.10 | 1.06±0.12 | 1.28±0.11 | 1.40±0.02 | 1.30±0.06 |
| C15:1n-5 | / | 0.07±0.01 | 0.07±0.02 | 0.07±0.03 | 0.10±0.02 | 0.12±0.02^*^ | 0.09±0.04 |
| C16:1n-7 | 1.67±0.03 | 1.26±0.25 | 0.89±0.23 | 1.13±0.14 | 0.83±0.07* | 0.99±0.34 | 0.74±0.09 |
| C18:1n-9c | 13.9±0.03 | 12.47±1.04^bc^ | 10.5±4.15^ab^ | 16.03±0.5^c^ | 10.32±1.68^b^ | 7.22±2.03^a^ | 5.89±0.80^a^ |
| C20:1n-9 | 1.67±0.02 | 1.42±0.05^a*^ | 1.59±0.10^ab^ | 1.63±0.13^ab^ | 1.93±0.25^b^ | 1.82±0.24^b^ | 1.66±0.07^ab^ |
| C22:1n-9 | 1.14±0.16 | 13.79±2.14^*^ | 10.5±2.24 | 9.57±1.07 | 13.73±4.00^*^ | 13.44±3.27 | 13.78±2.31 |
| C24:1 | 8.44±0.33 | 9.29±0.29^ab^ | 10.33±1.34^ab^ | 8.50±0.29^a^ | 10.17±0.16^ab^ | 10.76±0.73^b^ | 10.66±0.68 |
| C18:2n-6c | 1.83±0.02 | 2.32±0.17^c^ | 1.87±0.81^bc^ | 2.68±0.42^c^ | 1.37±0.53^ab^ | 1.02±0.52^ab^ | 0.45±0.04^a^ |
| C20:2 | / | 0.85±0.17^ab^ | 0.78±0.32^ab^ | 0.53±0.13^a^ | 0.74±0.02^ab^ | 1.42±0.95^b*^ | 2.43±0.22^c^ |
| C20:4n-6 | 17.21±0.08 | 17.6±0.32^a*^ | 22.59±4.63^b^ | 17.37±0.9^a^ | 21.65±0.70^ab^ | 22.62±1.87^b^ | 23.66±1.89^b^ |
| C22:2 | / | 0.17±0.03^ab^ | 0.13±0.05^ab^ | 0.10±0.02^a^ | 0.17±0.05^ab^ | 0.15±0.06^ab*^ | 0.20±0.05^b^ |
| C20:3n6 | 0.26±0.03 | / | / | / | / | / | / |
| C20:3n3 | 1.26±0.08 | / | / | / | / | / | / |
| C20:5n-3 | 11.74±0.02 | 3.62±0.98 | 3.42±0.11 | 3.04±0.37 | 2.84±0.43 | 4.03±0.20 | 2.94±0.27 |
| C22:6n-3 | 0.26±0.05 | / | / | / | / | / | / |
| SFA | 0.69±0.01 | 37.14±1.63^ab^ | 37.33±0.89^ab^ | 39.35±0.83^b^ | 36.16±0.74^a*^ | 36.42±1.07^ab^ | 37.50±0.87^ab^ |
| MUFA | 0.19±0.01 | 38.31±1.3^b*^ | 33.88±3.9^ab^ | 36.93±1.0^ab^ | 37.07±2.2^ab^ | 34.35±2.4^ab^ | 32.82±2.1^a^ |
| PUFA | 11.43±0.04 | 24.56±1.3^ab*^ | 28.79±4.1^bc^ | 23.72±0.2^a^ | 26.76±1.5^abc^ | 29.23±1.8^c^ | 29.68±2.3^c^ |
| TFA  (mg/100gDW) | 110.85±8.97 | 134.1±13.8^a^ | 133.5±2.4^a^ | 162.8±2.7^b^ | 121.5±5.1^a^ | 124.1±10.7^a^ | 112.1±13.2^a^* |


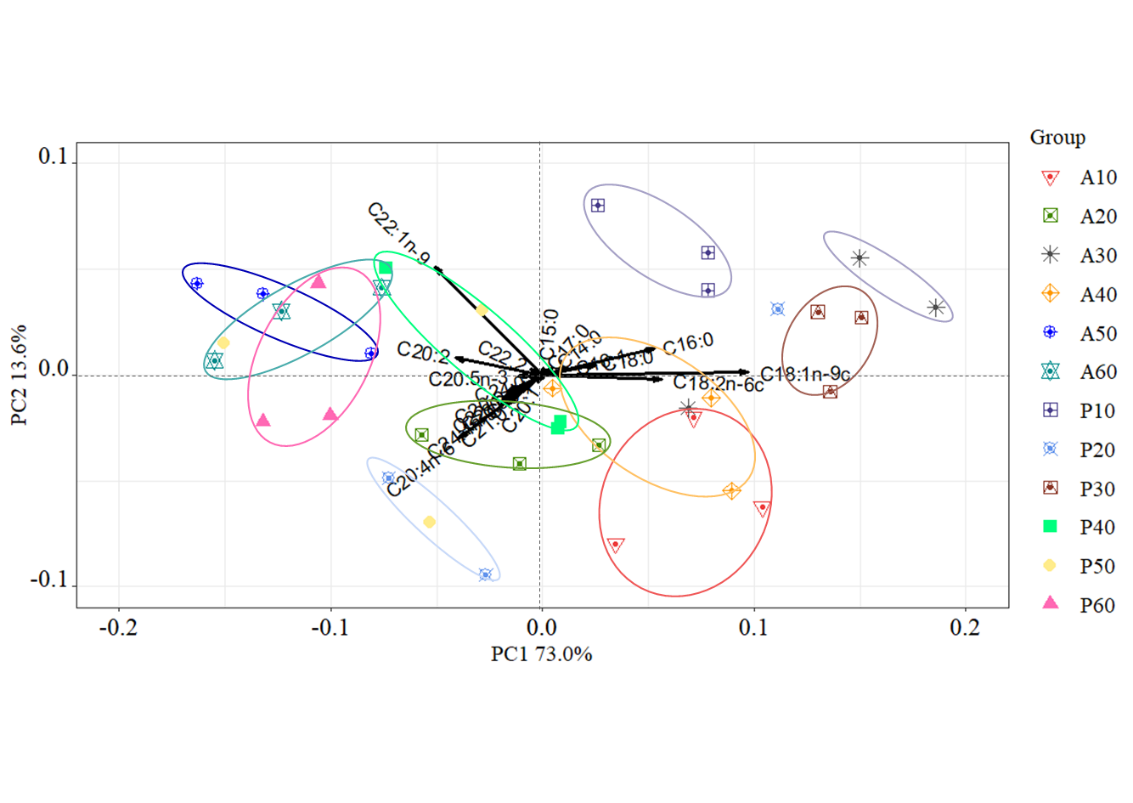


**Fig. S2** Principal component analysis of fatty acids (% of the total fatty acids) in two fission planes of sea cucumber *H. atra* over the regeneration course. Data was arcsine transformed. A10-A60 refers to the anterior part during the 10, 20, 30, 40, 50, and 60 regenerations, while P10-P60 refers to the posterior during the same regeneration period.

**Table S3** Principal component structure for the first two principal components (PC-1,-2) from a principal components analysis on fatty acid profile.

| Variable | PC-1 | PC-2 |
| --- | --- | --- |
| C18:1n-9 | 0.606 | -0.042 |
| C18:2n-6c | 0.348 | -0.073 |
| C16:0 | 0.336 | 0.087 |
| C18:0 | 0.157 | 0.027 |
| MUFA | 0.111 | 0.385 |
| SFA | 0.107 | 0.112 |
| C22:1n-9 | -0.297 | 0.724 |
| C20:4n-6 | -0.258 | -0.35 |
| C20:2 | -0.255 | 0.105 |
| PUFA | -0.237 | -0.293 |
